# Membrane transformations of fusion and budding

**DOI:** 10.1038/s41467-023-44539-7

**Published:** 2024-01-02

**Authors:** Ling-Gang Wu, Chung Yu Chan

**Affiliations:** National Institute of Neurological Disorders and Stroke, Bethesda, MD USA

**Keywords:** Membrane fusion, Endocytosis, Synaptic vesicle exocytosis

## Abstract

Membrane fusion and budding mediate fundamental processes like intracellular trafficking, exocytosis, and endocytosis. Fusion is thought to open a nanometer-range pore that may subsequently close or dilate irreversibly, whereas budding transforms flat membranes into vesicles. Reviewing recent breakthroughs in real-time visualization of membrane transformations well exceeding this classical view, we synthesize a new model and describe its underlying mechanistic principles and functions. Fusion involves hemi-to-full fusion, pore expansion, constriction and/or closure while fusing vesicles may shrink, enlarge, or receive another vesicle fusion; endocytosis follows exocytosis primarily by closing Ω-shaped profiles pre-formed through the flat-to-Λ-to-Ω-shape transition or formed via fusion. Calcium/SNARE-dependent fusion machinery, cytoskeleton-dependent membrane tension, osmotic pressure, calcium/dynamin-dependent fission machinery, and actin/dynamin-dependent force machinery work together to generate fusion and budding modes differing in pore status, vesicle size, speed and quantity, controls release probability, synchronization and content release rates/amounts, and underlies exo-endocytosis coupling to maintain membrane homeostasis. These transformations, underlying mechanisms, and functions may be conserved for fusion and budding in general.

## Abstract (web summary)

Life-crucial membrane fusion and budding were traditionally viewed with electron microscopy. With recent breakthroughs that visualize membrane transformation in real time, Wu and Chan synthesize a new model with mechanistic principles and functions.

## Introduction

**Fig. 1:**
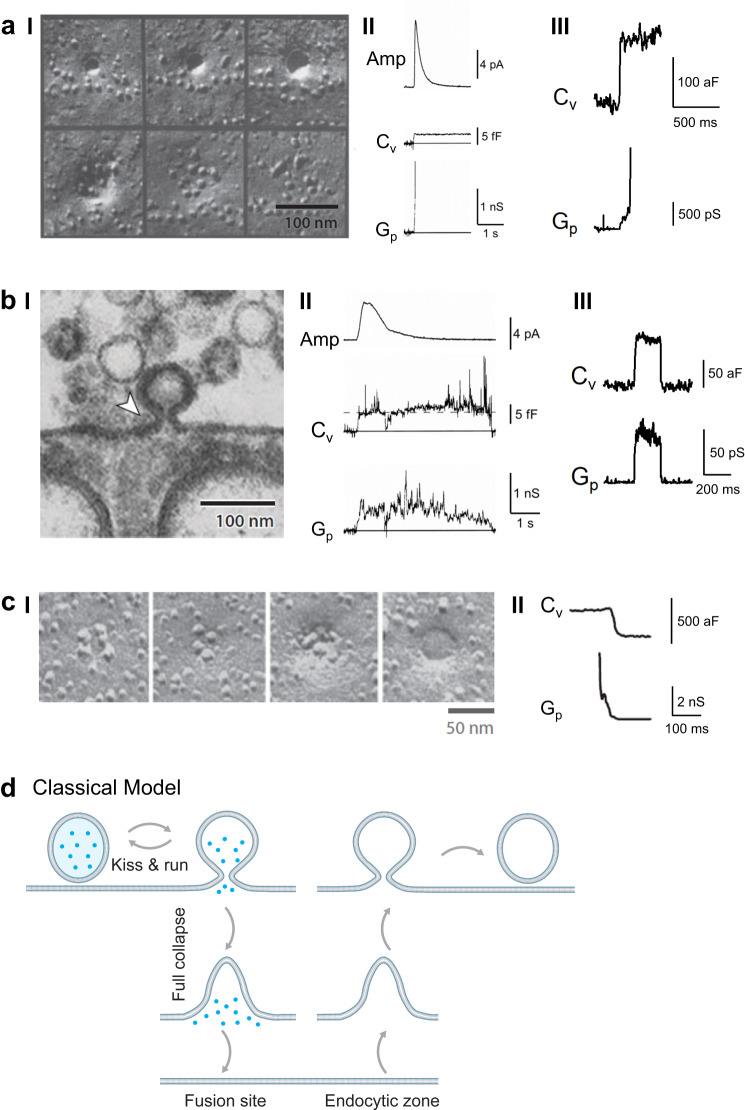
Classical exo-endocytosis model. **a** Full-collapse fusion. **I**, EM images showing the hypothesized sequence of full-collapse fusion (from upper left to upper right, then lower left to lower right). Images were taken from neuromuscular junctions that were frozen 3.7, 5.2, 5.2, 5.2, 20 ms, and 50 ms after stimulation. These data led to a full-collapse fusion proposal. Reproduced from J E Heuser, T S Reese; Structural changes after transmitter release at the frog neuromuscular junction. J Cell Biol 1 March 1981; 88 (3): 564–580. 10.1083/jcb.88.3.564. **II** Cell-attached recordings of amperometric current (Amp), vesicular membrane capacitance (*C*_v_), and fusion pore conductance (*G*_p_) at a chromaffin cell. Reproduced from Albillos, A., Dernick, G., Horstmann, H. et al. The exocytotic event in chromaffin cells revealed by patch amperometry. Nature 389, 509–512 (1997). 10.1038/39081. **III** Cell-attached capacitance recordings of *C*_v_ and *G*_p_ at the release face of a calyx of Held nerve terminal. Adapted from He, L., Wu, XS., Mohan, R. et al. Two modes of fusion pore opening revealed by cell-attached recordings at a synapse. Nature 444, 102–105 (2006). 10.1038/nature05250. **b** Kiss-and-run **I**. EM image of a Ω-shape profile in a frog neuromuscular junction led to kiss-and-run proposal. Reproduced from B. Ceccarelli, W. P. Hurlbut, A. Mauro; DEPLETION OF VESICLES FROM FROG NEUROMUSCULAR JUNCTIONS BY PROLONGED TETANIC STIMULATION. J Cell Biol 1 July 1972; 54 (1): 30–38. 10.1083/jcb.54.1.30. **II** Cell-attached recordings of Amp, C_v,_ and G_p_ at a chromaffin cell. Reproduced from Albillos, A., Dernick, G., Horstmann, H. et al. The exocytotic event in chromaffin cells revealed by patch amperometry. Nature 389, 509–512 (1997). 10.1038/39081. **III** Cell-attached recordings of *C*_v_ and *G*_p_ during a capacitance flicker at the release face of a calyx of Held nerve terminal. Adapted from He, L., Wu, XS., Mohan, R. et al. Two modes of fusion pore opening revealed by cell-attached recordings at a synapse. Nature 444, 102–105 (2006). 10.1038/nature05250. **c** Endocytic membrane transformations. **I**. Freeze-fracture EM images of frog neuromuscular junctions arranged in this order to illustrate the hypothetical endocytic process starting from a shallow pit to a vesicle-like Ω-profile. Reproduced from T M Miller, J E Heuser; Endocytosis of synaptic vesicle membrane at the frog neuromuscular junction. J Cell Biol 1 February 1984; 98 (2): 685–698. 10.1083/jcb.98.2.685. **II** Cell-attached recordings of *C*_v_ and *G*_p_ reflecting fission pore closure at a calyx of Held release face. Adapted from He, L., Xue, L., Xu, J. et al. Compound vesicle fusion increases quantal size and potentiates synaptic transmission. Nature 459, 93–97 (2009). 10.1038/nature07860. **d** Classical exo-endocytosis model. Fusion undergoes kiss-&-run or full-collapse fusion, the latter of which is followed by endocytic flat-to-round transformation at an endocytic zone. Blue dots: vesicular contents before fusion (applies to all figures).

Membrane fusion and budding mediate fundamental biological processes, such as neurotransmitter and hormone release crucial for brain functions, intracellular trafficking, formation of membrane-bound organelles (e.g., vesicles, exosomes, and mitochondria), fertilization, vesicle recycling, nutrient uptake, cell fusion and division, and viral infection^1–3^. Current understanding of their membrane transformations is mainly derived from exo- and endocytosis studies in neurons and endocrine cells. Half a century of studies (Fig. 1a–c) established the classical exo-endocytosis framework (Fig. 1d)—vesicle fusion at the plasma membrane (PM) opens a narrow (<~5 nm) pore, which may close rapidly to limit vesicular content release, called kiss-and-run, or dilate until the vesicle flattens to promote release, called full-collapse fusion; subsequent flat-to-round endocytic membrane transformation retrieves fused vesicles^2,4–9^. Studies of exo-endocytosis and fusion budding in general have been interpreted under this view, which has not been verified by real-time observation in live cells^2,7,10,11^.

**Fig. 2:**
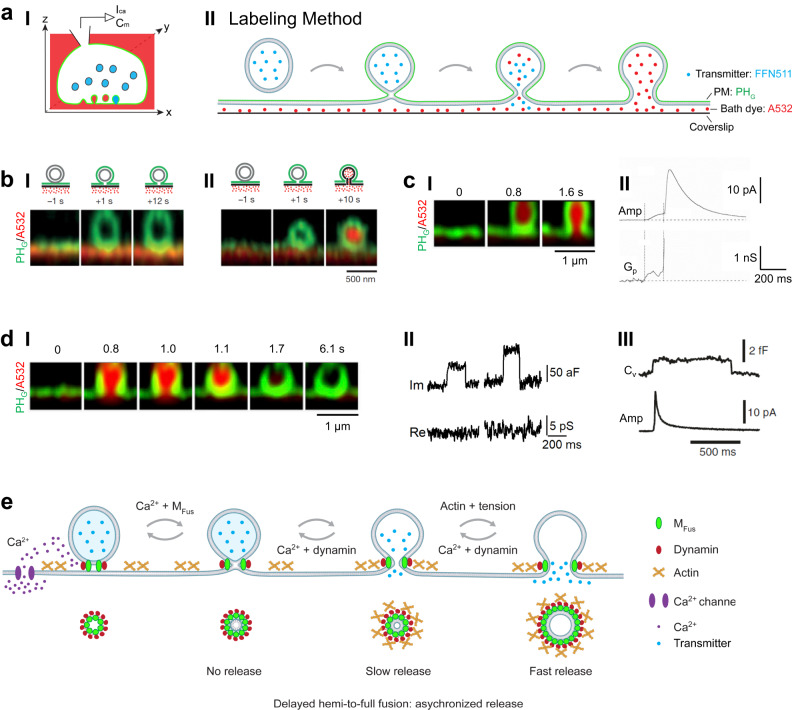
A dynamic pore model of fusion. **a** Schematic diagram for setup and labeling method. **I** In a chromaffin cell, vesicles are preloaded with FFN511 (blue), PM inner leaflet labeled with overexpressed PH_G_ (green), extracellular solution labeled with A532 (red), a pipette at the whole-cell voltage-clamp configuration for delivering depolarization and recording calcium currents (*I*_Ca_) and membrane capacitance (*C*_m_). **II** Fusion dynamics (hemi-fusion, pore opening and expansion) are visualized with three probes: FFN511 for release, PH_G_ for PM inner leaflet fusion, and A532 for A532-permeable pore. **b** STED XZ plane images of PH_G_ (green) and A532 (red) showing hemi-fusion (**I**) and hemi-fusion followed by hemi-to-full fusion (**II**). Images taken at times relative to 1-s depolarization as labeled (also applies to **cI** and **dI**). Adapted from Zhao, WD., Hamid, E., Shin, W. et al. Hemi-fused structure mediates and controls fusion and fission in live cells. Nature 534, 548–552 (2016). 10.1038/nature18598. **c** Fusion pore expansion. **I** STED XZ plane images of PH_G_ and A532 showing fusion pore opening (Middle, A532-permeable) and expansion (right, visible). Data taken from *Cell*, **173**, W. Shin, L. Ge, G. Arpino, et al. Visualization of Membrane Pore in Live Cells Reveals a Dynamic-Pore Theory Governing Fusion and Endocytosis, 934-945, Copyright Elsevier (2018). **II** Cell-attached recordings of the amperometric current (Amp) and fusion pore conductance (G_P_). Reproduced from Albillos, A., Dernick, G., Horstmann, H. et al. The exocytotic event in chromaffin cells revealed by patch amperometry. Nature 389, 509–512 (1997). 10.1038/39081. **d** Fusion pore closure (kiss-and-run). **I** STED XZ plane images of PH_G_ and A532 showing fusion pore opening and closure. Pore closure prevents bleached A532 from exchange with fluorescent A532 in the bath, leading to A532 fluorescence dimming. Data were published in *Cell*, **173**, W. Shin, L. Ge, G. Arpino, et al. Visualization of Membrane Pore in Live Cells Reveals a Dynamic-Pore Theory Governing Fusion and Endocytosis, 934-945, Copyright Elsevier (2018). **II** Capacitance (Im) flickers with pore conductance (Re) beyond detection limit from cell-attached recordings at the calyx of Held release face Adapted from He, L., Wu, XS., Mohan, R. et al. Two modes of fusion pore opening revealed by cell-attached recordings at a synapse. Nature 444, 102–105 (2006). 10.1038/nature05250. **III** Cell-attached recordings of a capacitance flicker with a fast amperometric spike at a chromaffin cell. Reproduced from Alés, E., Tabares, L., Poyato, J. et al. High calcium concentrations shift the mode of exocytosis to the kiss-and-run mechanism. Nat Cell Biol 1, 40–44 (1999). 10.1038/9012. **e** Schematics of fusion pore dynamics and underlying mechanisms. Fusion undergoes hemi-fusion, hemi-to-full fusion, pore expansion, constriction and closure. The dynamics of each of these transitions are the net outcome of competition between fusion machinery (M_Fus_), plasma membrane tension and dynamin. Calcium influx triggers vesicle fusion, fusion pore constriction and closure. Hemi-fusion generates no release; small and large fusion pore generates slow and fast release, respectively. Delayed hemi-to-full fusion causes asynchronized release.

**Fig. 3:**
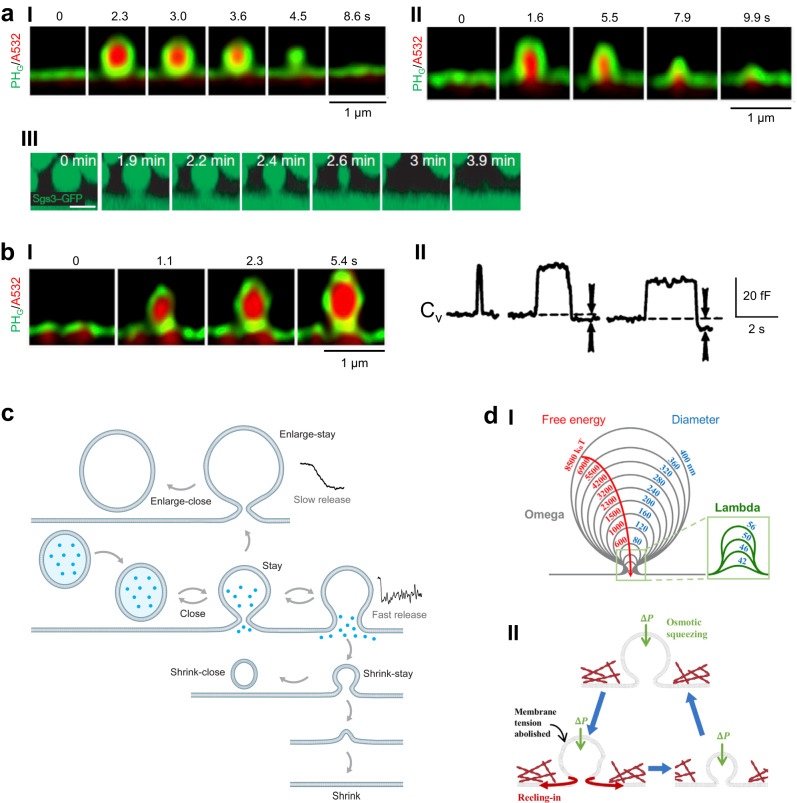
Seven fusion modes differed in pore status and vesicle size. **a** Shrink fusion. **I–II** STED XZ plane images of PH_G_ (green) and A532 (red) showing shrink fusion: fusion-generated Ω-profiles may shrink until undetectable (**I**), or until becoming a Λ-shape at the very end of the shrinking process (**II**). Images taken at times labeled. Data were published in *Cell Report*, **30**, W. Shin, G. Arpino, S. Thiyagarajan, et al. Vesicle Shrinking and Enlargement Play Opposing Roles in the Release of Exocytotic Contents, 421–431, Copyright Elsevier (2020). **III**. Confocal XZ plane images showing a huge GFP-filled salivary gland vesicle undergoing shrinking at times labeled. Scale bar: 5 µm. Reproduced from Rousso, T., Schejter, E. & Shilo, BZ. Orchestrated content release from Drosophila glue-protein vesicles by a contractile actomyosin network. Nat Cell Biol 18, 181–190 (2016). 10.1038/ncb3288. **b** Enlarge fusion. **I** STED XZ plane images of PH_G_ (green) and A532 (red) showing enlarge fusion: the fusion-generated Ω-profile enlarged over time as labeled. Data were published in *Cell Report*, **30**, W. Shin, G. Arpino, S. Thiyagarajan, et al. Vesicle Shrinking and Enlargement Play Opposing Roles in the Release of Exocytotic Contents, 421-431, Copyright Elsevier (2020). **II** Capacitance flickers with the down-step equal to (left) or larger than (middle and right) the up-step. Reproduced from J.R. Monck, T.G. Alvarez de, J.M. Fernandez, Tension in secretory granule membranes causes extensive membrane transfer through the exocytotic fusion pore. *Proc. Natl. Acad. Sci. USA* **87**, 7804–7808 (1990). Middle and right panel are consistent with enlarge-close fusion. **c** Schematic diagram describing seven fusion modes: stay, close, enlarge-stay, enlarge-close, shrink-stay, shrink-close, and shrink fusion. Shrink-related fusion events are associated with a larger pore to generate fast release; enlarge-related fusion events are associated with a smaller pore to generate slow release. Release traces are taken from the article published in *Cell Report*, **30**, W. Shin, G. Arpino, S. Thiyagarajan, et al. Vesicle shrinking and enlargement play opposing roles in the release of exocytotic contents, 421-431, Copyright Elsevier (2020). **d I** Predicted shrink fusion sequence. Computed vesicle shapes and free energies for squeezing pressure Δ*P* = 100 Pa and the indicated effective vesicle diameter (D). A transition from Ω- to Λ-shape occurs at *D* = 56 nm. Data were published in *Cell Report*, **30**, W. Shin, G. Arpino, S. Thiyagarajan, *et al*. Vesicle Shrinking and Enlargement Play Opposing Roles in the Release of Exocytotic Contents, 421-431, Copyright Elsevier (2020). **II** Schematic diagram showing the osmotic pressure difference between the intracellular and the extracellular solution (ΔP) squeezes and thus deflates the vesicle, abolishing the vesicular membrane tension and allowing for the vesicular membrane to be reeled into the plasma membrane by the high plasma membrane tension and the actin cortex. Drawing taken from the article published in *Cell Report*, **30**, W. Shin, G. Arpino, S. Thiyagarajan, et al. Vesicle Shrinking and Enlargement Play Opposing Roles in the Release of Exocytotic Contents, 421–431, Copyright Elsevier (2020).

**Fig. 4:**
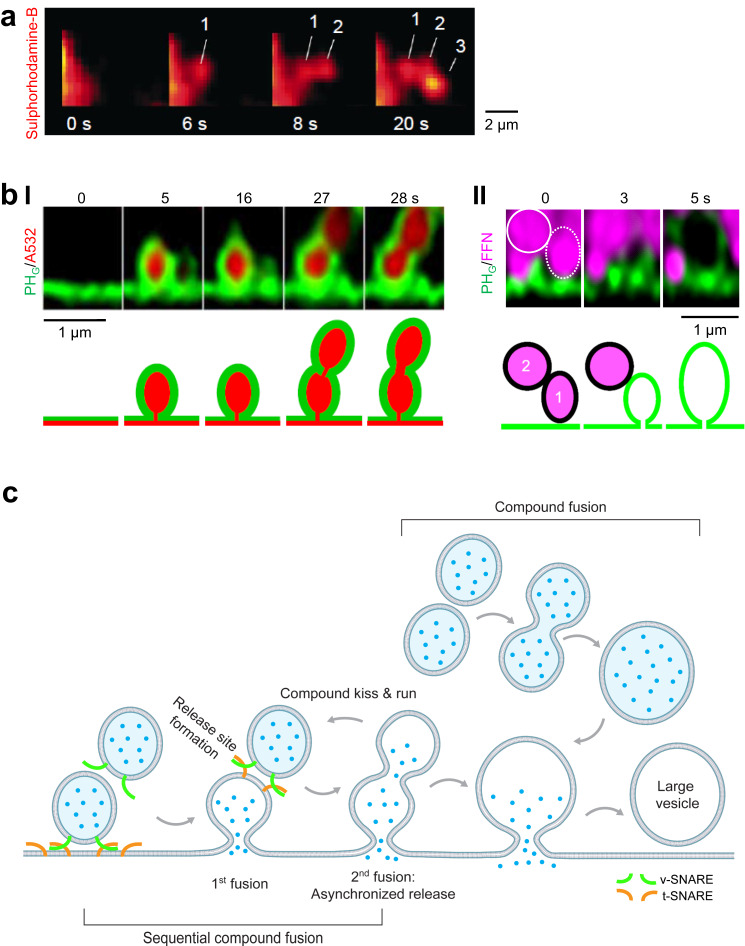
Sequential compound fusion. **a** Three sulphorhodamine-B-filled spots (red) occurred sequentially at the indicated time that may reflect sequential compound fusion of zymogen granules in a pancreatic acinus cell bathed with sulphorhodamine-B. Reproduced from Nemoto, T., Kimura, R., Ito, K. et al. Sequential-replenishment mechanism of exocytosis in pancreatic acini. Nat Cell Biol 3, 253–258 (2001). 10.1038/35060042. **b** Sequential compound fusion and release visualized in chromaffin cells. **I** STED XZ plane images of PH_G_ (green) and A532 (red) showing sequential compound fusion at times indicated from a chromaffin cell (see also cartoon explanations). Adapted from L. Ge, W. Shin, G. Arpino, L. Wei, C. Y. Chan, C. K. E. Bleck, W. Zhao, L. G. Wu, Sequential compound fusion and kiss-and-run mediate exo- and endocytosis in excitable cells. *Sci. Adv*. **8**, eabm6049 (2022). © The Authors, some rights reserved; exclusive licensee AAAS. Distributed under a Creative Commons Attribution NonCommercial License 4.0 (CC BY-NC) http://creativecommons.org/licenses/by-nc/4.0/. **II**. STED XZ plane images of PH_G_ (green) and FFN511 (magenta) showing sequential compound release (two circles) at times indicated from a chromaffin cell (see also cartoon explanations). Adapted from L. Ge, W. Shin, G. Arpino, L. Wei, C. Y. Chan, C. K. E. Bleck, W. Zhao, L. G. Wu, Sequential compound fusion and kiss-and-run mediate exo- and endocytosis in excitable cells. *Sci. Adv*. **8**, eabm6049 (2022). © The Authors, some rights reserved; exclusive licensee AAAS. Distributed under a Creative Commons Attribution NonCommercial License 4.0 (CC BY-NC) http://creativecommons.org/licenses/by-nc/4.0/. **c** Schematic diagram depicting membrane transformations of sequential compound fusion, sequential compound kiss-and-run, and compound fusion. New release site formation at the 1st fused vesicle enables sequential compound fusion; 2nd fusion generates asynchronized release; sequential compound fusion or compound fusion may generate large Ω-profiles and vesicles.

**Fig. 5:**
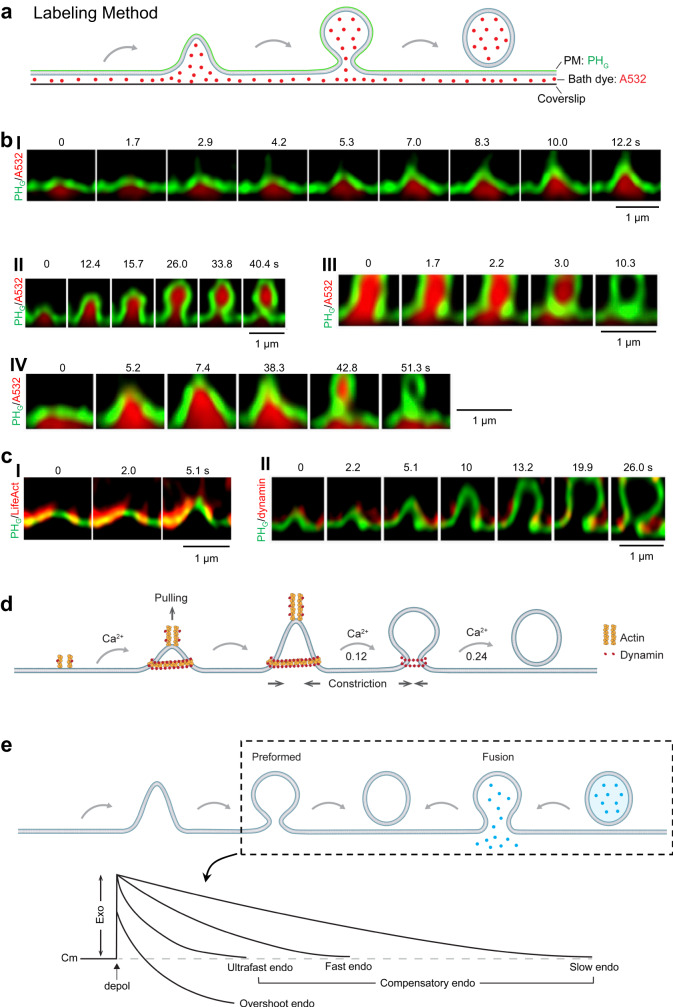
Endocytic membrane transformation, underlying mechanical forces, and function in generating diverse endocytic modes. **a** Schematic diagram showing how to visualize endocytic membrane transformation using two probes: PH_G_ (green) for labeling the PM inner leaflet and A532 (red) for labeling bath solution (bath dye). **b** STED XZ plane images of PH_G_ (green) and A532 (red) showing Flat→Λ (I), Λ→Ω (II), Ω→O (III) and Flat→Λ→Ω→O transition (IV). Images taken at times labeled. Data in panel I were adapted from Shin, W., Zucker, B., Kundu, N. et al. Molecular mechanics underlying flat-to-round membrane budding in live secretory cells. Nat Commun 13, 3697 (2022). 10.1038/s41467-022-31286-4. Data in panels II-IV were from the article published in *Neuron*, **109**, W. Shin, L. Wei, G. Arpino, et al. Preformed Omega-profile closure and kiss-and-run mediate endocytosis and diverse endocytic modes in neuroendocrine chromaffin cells, 3119–3134, Copyright Elsevier (2021). **c** **I** STED XZ plane images of PH_G_ (green) and Lifeact-mTFP1 (red, labeling F-actin) showing spike-like PH_G_-labeled membrane protrusion attached to growing F-actin filaments while the Λ-profile is growing. Adapted from Shin, W., Zucker, B., Kundu, N. et al. Molecular mechanics underlying flat-to-round membrane budding in live secretory cells. Nat Commun 13, 3697 (2022). 10.1038/s41467-022-31286-4. **II** STED XZ plane images of PH_G_ (green) and dynamin-mTFP1 (red) showing dynamin puncta flanked and moved with constricting Λ’s base and constricting Ω’s pore—dynamin constricts Λ’s base and Ω’s pore. Adapted from Shin, W., Zucker, B., Kundu, N. et al. Molecular mechanics underlying flat-to-round membrane budding in live secretory cells. Nat Commun 13, 3697 (2022). 10.1038/s41467-022-31286-4. **d** Schematic diagram showing the Flat→Λ→Ω→O transition mediated by two forces, the F-actin- and dynamin-dependent pulling force and dynamin-dependent constriction force. Calcium is the trigger for each transition, including Flat→Λ, Λ→Ω and Ω→O. The probability of each transition is low as labeled (0.12–0.24). **e** Pore closure of preformed Ω-profiles and fusion pores, but not flat-to-round transformation, is the main driving force underlying diverse modes of endocytosis, such as ultrafast, fast, slow, compensatory, and overshoot endocytosis that follow depolarization-induced exocytosis. Depol: depolarization; Cm: membrane capacitance, Exo: exocytosis (Cm increase); endo: endocytosis (Cm decay); blue dots: vesicular contents before fusion.

**Fig. 6:**
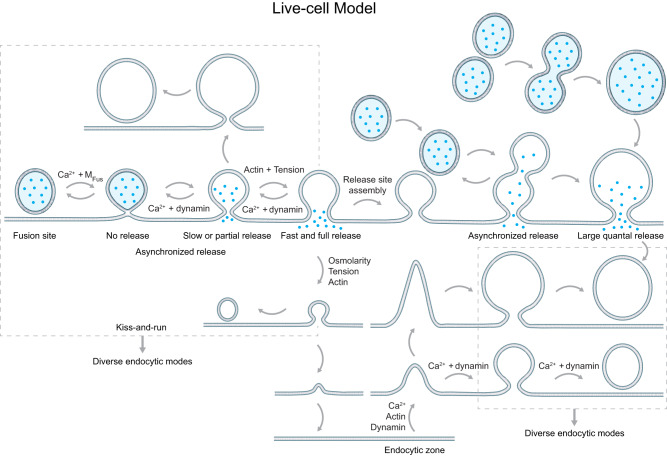
A new exo-endocytosis membrane transformation model synthesized from live-cell observations. A schematic diagram showing a new exo-endocytosis membrane transformation model and molecular mechanical mechanisms underlying each membrane transition (*M*_Fus_: fusion machinery). Hemi-fusion generates no release (blue dots: vesicular contents); enlarge-related fusion generates slow release; shrink-related fusion generates fast release. Delayed hemi-to-full fusion or sequential compound fusion generates asynchronized release. Compound fusion generates a large quantal size. Narrow fusion pore may cause partial vesicular content release. Redefined kiss-and-run (including close, enlarge-close and shrink-close fusion, left dash square) and preformed Ω-profile pore closure (right dash square) are major mechanisms underlying diverse endocytic modes, including ultrafast, fast, slow, compensatory, overshoot, and bulk endocytosis.

Recent super-resolution microscopy visualized exo-endocytosis membrane dynamics in live neuroendocrine cells. Novel membrane transformations, underlying mechanisms, and functions far exceeding the classical view have emerged. Here, we review visualization methods, real-time-observed membrane transformations, underlying molecular mechanics, and functions in governing exo-endocytosis (Figs. 2–5). We synthesize a new fusion-budding membrane dynamics framework (Fig. 6), replacing the classical framework as the new platform for building a future comprehensive molecular model and reinterpreting previous studies working under the classical view.

## Classical exo-endocytosis framework

Electron microscopic (EM) observation of pore-like structures at nerve terminals milliseconds after stimulation led to the full-collapse fusion hypothesis (Fig. 1aI)^6^. Full-collapse fusion was thus used to interpret the widely observed abrupt fusion pore conductance increases beyond detection limit (estimated as ~5 nm)^12–15^, fast synaptic currents, and fast catecholamine-generated amperometric currents in secretory cells (Fig. 1aII–III)^2,7,13,15,16^. An equally possible interpretation—large pores without collapse—was neglected. EM observation of Ω-profiles led to the kiss-and-run proposal (Fig. 1bI)^5^. Subsequent observations at live synapses and endocrine cells support the kiss-and-run proposal: (1) capacitance flickers (equal up- and down-steps) reflecting single vesicular membrane addition and subtraction were sometimes accompanied by a detectable pore conductance or a slower amperometric current (Fig. 1bII–III)^13,15,17^, and (2) failure in releasing quantum dots or proteins from fusing vesicles^2,7,18–20^.

Endocytic vesicle formation may undergo a flat-to-round transformation. This concept is supported by EM observation of shallow and deep membrane pits (Fig. 1cI)^4,21^, and detection of pore conductance decreases reflecting fission, the final step of endocytosis, in live synapses (Fig. 1cII)^22,23^.

Full-collapse fusion followed by endocytic flat-to-round transition is considered the primary exo-endocytosis mode. In contrast, kiss-and-run, despite receiving stronger support in live cells, had been questioned and considered at most a minor mode ever since its proposal^2,7,24^. Real-time visualization of these modes is needed to establish the classical exo-endocytosis framework (Fig. 1d).

## A system for visualizing fusion and budding

Fusion and budding are most studied for small ~30–80 nm synaptic vesicles and ~40–100 nm clathrin-coated vesicles. Various super-resolution microscopies with stochastic optical reconstruction or photoactivated localization, structured illumination, stimulated emission depletion (STED), or minimal photon flux have led to nanoscale insights into exo- and endocytic sites, protein composition, protein localization, nanocolumn structures, and single molecule dynamics at nerve terminals^25–38^. However, rapid membrane transformations of ~30–100 nm vesicles have not been visualized owing to insufficient spatial-temporal resolution.

To visualize membrane transformation regularly requires abundant, synchronized exo-endocytosis events imaged at sub-vesicle and sub-second resolution. These criteria were met by STED microscopy at ~60–80 nm resolution every ~26–300 ms in live bovine adrenal chromaffin cells in primary culture, where tens to hundreds of ~200–1500 nm vesicle exo-endocytosis are induced within seconds after a 1-s depolarization (Fig. 2a, Box 1)^39–42^. With fluorescent probes labeling PM cytosol-facing leaflet, extracellular-facing leaflet, extracellular solution, vesicular lumen contents, and/or exo-endocytosis proteins, 2- and 3-color STED microscopy recently real-time visualized exo-endocytosis membrane transformations and proteins driving these transformations (Fig. 2a, Box 1). For example, in cells bathed with Atto 532 (A532) and overexpressed with phospholipase C ΔPH domain attached with mNeonGreen or EGFP (PH_G_), which binds PtdIns(4,5)P_2_ at, and thus labels, PM cytosol-facing leaflet, PH_G_ and A532 diffusion into fusing or endocytic structures allows for imaging membrane transformations (Fig. 2b–d, Supplementary Movies 1-3). For fusing or endocytic Ω-profile’s pores below STED resolution, permeation of small fluorescent molecules like A532 is used to indicate an opened pore (Fig. 2b, c; Box 1)^40^. These labeling strategies can be used to visualize fusion and budding in general.

Box 1. A system for visualizing exo-endocytosis membrane dynamics STED microscopy at ~60–80 nm resolution per 26–300 ms has visualized exo- and endocytosis membrane transformations in live adrenal chromaffin cells^39–42^. Three categories of fluorescent probes are used to label (1) the PM cytosol-facing leaflet (PM_cyto_) or extracellular-facing leaflet (PM_extra_) that may indicate membrane transformations, (2) the bath solution that may permeate into, and thus indicate pore status of, fusing or budding vesicles, and (3) vesicular lumen contents that may indicate their release upon fusion pore opening (Fig. 2a)^39–42^. PM_cyto_ can be labeled by overexpressing mNeonGreen (or other fluorescent proteins) tagged phospholipase C δ1 PH domain (PH_G_), which binds to PtdIns(4,5)P_2_^195^ at PM_cyto_, or fluorescently tagged Lyn kinase’s myristoylation and palmitoylation sequence or CAAX^39^ (Fig. 2a). Addition of mCLING-Atto 488 (mCLING: membrane-binding fluorophore-cysteine-lysine-palmitoyl group) in the bath solution allows mCLING-Atto 488 to insert into, and thus label PM_extra_^39,196^ (Fig. 2a). Bath solution can be labeled by adding a cell-impermeable dye, such as Atto 532 or Atto 655 (Fig. 2a). Vesicular lumen contents can be labeled by overexpressing fluorescently tag vesicular lumen proteins (e.g., neuropeptide Y-EGFP) or adding fluorescent false neurotransmitter (e.g., FFN511) into the bath, which can be taken up into vesicles^197^.

Upon vesicle hemi-fusion or full fusion with PM, PM_cyto_-labeling probes, such as PH_G_, diffuse into, and thus label, the hemi-fused or fully fused vesicular cytosol-facing leaflet (Fig. 2a–d). Upon hemi-to-full fusion, PM_extra_-labeling probes, such as mCLING-Atto 488, diffuse into, and thus label, the fully fused vesicular extracellular-facing leaflet^39^; bath-solution-labeling probes, such as Atto 532, diffuse via the fusion pore to label the fully fused vesicle cavity (Fig. 2a–d); vesicular lumen-labeling probes, such as FFN511, are released via the opened fusion pore (Fig. 4bII). Similarly, PM-labeling probes indicate endocytic flat-to-round membrane transformation, whereas bath-solution-labeling probes indicate the permeability of the endocytic structure to the bath solution (Fig. 5a–c).

With the above fluorescent probes, STED microscopy visualizes hemi-fusion, hemi-to-full fusion, fusion pore dynamics, and endocytic flat-to-round membrane transformation (Figs. Fig. 2b–d, 3a, b, 4b, 5b, c). To observe fast membrane dynamics, images are mostly scanned at a single microscopic XZ plane (e.g., Fig. 2a–c), which reduces the chance of seeing fusion or endocytic transitions. To increase the chance, a brief (e.g., 1 s) depolarization via a pipette at the whole-cell voltage-clamp configuration is applied, which may induce within seconds up to hundreds of exo-endocytosis events in a cell, allowing for seeing exo-endocytosis events before significant bleaching of fluorescent probes or phototoxicity occurs.

One pitfall of STED imaging is its inability to resolve Ω-profiles’ pores below its resolution, ~60 nm. However, Ω-profiles’ permeation to bath-solution-labeling probes like Atto 532 can be used to detect an open pore (e.g., Fig. 2b, c). With strong excitation of the bath-solution-labeling probes Ω-profile pore closure is detected as fluorescence dimming because pore closure prevents bath fluorescent molecules from exchanging with bleached molecules inside the closed Ω-profile (e.g., Figs. 2dI, 5bIII, IV)^39,40,54,66^. This method detects dynamin-dependent pore closures impermeable to H+ and OH-, coinciding with the PH_G_-labeled pore closure directly observed with STED imaging (Fig. 2dI)^39,40,54,66^.

## Dynamic membrane transformations

### Exocytotic membrane transformation

#### The hemi-fusion pathway in live cells

Hemi-fusion, the fusion between proximal leaflets of two fusing membrane bilayers, was proposed as the pathway to full fusion^43–45^. A competing hypothesis with a protein-lined pore made of SNARE proteins as the initial fusion product was also proposed^46–49^. Distinguishing these hypotheses was once considered impractical. Recent imaging in live cells visualized the hemi-fusion structure as a PH_G_-labeled Ω-profile (labeling cytosol-facing leaflet) impermeable to small molecules like A532 and H^+^ (Fig. 2aI, Supplementary Movie 1), and impermeable to probes labeling the extracellular-facing leaflet^39^. A surprisingly large fraction (~30–40%) of fusion undergoes hemi-fusion with a detectable lifetime, among which one-third undergoes hemi-to-full fusion within ~0.1–26 s (Fig. 2bII, Supplementary Movie 2), and the remaining may undergo hemi-to-full fission^39^. Excluding the pure protein-lined pore hypothesis, these results revealed the hemi-fusion pathway in live cells. However, SNARE proteins might form parts of the fusion pore, as SNARE proteins were found to be exposed to polar solvents during fusion in nano-disks^50^.

#### Fusion pore opening dynamics

Conductance measurements may derive fusion pores less than ~5 nm within milliseconds under the assumption of a fixed pore geometry^13,15,17,23,51,52^. In contrast, STED microscopy visualizes pores directly, but only for large pores (~60 nm) at a slow speed (every 26-300 ms): ~180–720 nm vesicles open a ~0–490 nm pore (Fig. 2c–e); these pores may expand rapidly at >~9 nm/ms to support fast content release (Fig. 2d, Supplementary Movie 3); some pores are initially small (<~60 nm) for ~0.5–4 s, but may expand abruptly (Fig. 2c)^40^. These results visualized fusion pore expansion implicated from recordings of small pore conductance preceding an abrupt increase (Fig. 1aIII) or slow amperometric foot signal preceding a fast spike (Fig. 2cII)^12,13,53^. The maintenance of the Ω-shape with large pores (Fig. 2c, d)^40^ indicates that traditional interpretation of abrupt pore conductance increase and/or fast amperometric spike as full-collapse fusion should not be practiced.

#### Seven fusion modes regarding Ω-profile size and pore

Fusion-generated, PH_G_-labeled Ω-profiles may remain unchanged (same size, Fig. 2b–d), shrink partially or completely within seconds (Fig. 3a, Supplementary Movie 4), or enlarge (Fig. 3b, Supplementary Movie 5), while its pore may stay open or close^40,41,54^. Accordingly, there are seven modes reflecting Ω-profile size and pore status (Fig. 3c): enlarge-stay (enlarged, pore opened), enlarge-close (enlarged, pore closed), stay (same size, pore opened), close (same size, pore closed), shrink-stay (partially shrink, pore opened), shrink-close (partially shrink, pore closed), and shrink fusion (Ω-profile shrinks completely)^40,41,54^. Surprisingly, full-collapse fusion, the primary fusion mode generally thought^40,41,55,56^, was not observed.

These fusion modes are common but have not been fully recognized previously. For example, capacitance up-steps reflecting single vesicle fusion were sometimes followed by larger down-steps in mast cells (Fig. 3bII)^57^, or smaller down-steps at nerve terminals^58^, supporting enlarge-close and shrink-close fusion, respectively. Unequal capacitance up- and down-steps observed at calyx nerve terminals^15,23,59^ and likely many other cell types could be caused by, but had not been interpreted as, shrink-close or enlarge-close fusion. Extremely large vesicles (~1–10 μm) may shrink in minutes in exocrine cells (Fig. 3aIII)^55,56^, consistent with shrink or shrink-stay fusion.

#### Redefining kiss-and-run

Fusion pores up to ~490 nm may remain open or constrict and then close (Fig. 2cI, dI) while Ω-profile size may change (Fig. 3a, c)^40,41,54^. The traditionally defined kiss-and-run, “closure of narrow (<~5 nm) pores to limit content release, but form vesicles identical to original ones” (Fig. 1d)^2,7^, should be redefined as “closure of fusion pores of any size that may limit or promote content release (Fig. 2e), and may form vesicles of different sizes (Fig. 3c). Supporting this redefinition, capacitance flickers are often accompanied by a too-large-to-detect conductance at synapses (Fig. 2dII)^15^ and fast amperometric spikes in endocrine cells (Fig. 2dIII);^13,14^ unequal capacitance up- and down-steps consistent with shrink-close or enlarge-close fusion were reported^15,57,58^.

#### Debate between shrink and full-collapse fusion

Shrink fusion was observed down to ~60 nm^41^, suggesting that ~30–80 nm synaptic vesicles might undergo shrink fusion. Imaging and modeling showed that at the final stage of shrinking, Ω-shape is converted to Λ-profile (Fig. 3aII, dI). Accordingly, a shrink-collapse mode, in which Ω-profile shrinking is followed by Ω-to-Λ-to-flat shape transition, is suggested to reconcile the conflict (Fig. 3c): shrinking is dominant when the fusing Ω-profile size is large, ~60–80 nm, whereas full-collapse (Ω-to-Λ-to-flat) may become dominant as the shrinking Ω-profile size reaches ~30–10 nm^41^.

#### Sequential compound fusion and kiss-and-run

Fusion at a previously fused vesicle, termed sequential compound fusion, was proposed for large (~1–10 μm) exocrine vesicles that release contents very slowly (Fig. 4a)^60–62^. STED microscopy of smaller endocrine vesicles observed and thus proved the proposed membrane transformation and revealed its pore dynamics (Fig. 4b, c)^42^. Sequential compound fusion forms an 8-shape structure with a pore that may (1) dilate to form a larger and elongated Ω-profile (Fig. 4bI-II; Supplementary Movies 6 and 7), (2) close to recycle vesicles, termed sequential compound kiss-and-run, or (3) remain unchanged (Fig. 4c)^42^.

#### Compound fusion

Compound fusion, the vesicle-vesicle fusion that forms larger vesicles for subsequent exocytosis (Fig. 4c)^2^, is supported by (1) EM observation of multivesicular-shaped vesicles, vesicles much larger than regular ones, and large vesicular-shape structures at secretory cell PM, (2) capacitance up-steps and miniature excitatory postsynaptic currents reflecting single synaptic vesicle fusion larger than regular ones, and (3) capacitance up-steps equivalent to several vesicles’ membrane capacitances accompanied by the release of multiple fluorescently labeled vesicles in eosinophils^2,23,60,62–65^. Compound fusion’s membrane transformation has not been real-time visualized.

### Endocytic membrane transformation

#### Three modular transformations: flat→Λ, Λ→Ω, and Ω→Ο

Shallow and deep pits are considered intermediates of endocytic flat-to-round transition (Fig. 1cI)^4,21^, but difficult to prove with EM. STED imaging observed three modular transitions that formed ~200–1500 nm vesicles after depolarization-induced exocytosis: flat→Λ-shape, Λ→Ω-shape and Ω→Ο-shape, each of which takes 0.05-51 s (Fig. 5a–e; Supplementary Movies 8–11)^66^. Owing to low transition probabilities (~0.12–0.24), flat→Λ and Λ→Ω may not necessarily continue their transition towards Ο-shape (Fig. 5b)^66^, leaving many Λ- (or dome-shape) and Ω-profiles at PM. Upon subsequent depolarization, these preformed Λ- and Ω-profiles may continue their endocytic journey (Fig. 5e).

#### Preformed-Ω→Ο as the main driving force

Low probability Λ→Ω and Ω→Ο transition make flat→Ο transition much less frequent than preformed-Ω→Ο transition^66^. Furthermore, flat→Ο takes more steps and time than preformed-Ω→Ο^66^. Endocytic vesicle formation is thus primarily from preformed-Ω→Ο, but not the generally assumed flat→Ο (Fig. 5e)^66^. This ‘genius’ design fulfils physiological demands by offering much faster and more endocytosis from an apparently ‘clumsy’ machinery that drives flat→Λ→Ω→Ο slowly at low probabilities.

#### Endocytic zones are separated from fusion sites

The general belief that endocytosis occurs at endocytic zones different from release sites^4,21^ has only been visualized recently in chromaffin cells, where flat→Λ→Ω→Ο transitions occur at sites separated from fusion sites^40,66^. Mechanisms that separate release from endocytosis and generate endocytic zones remain poorly understood.

## Membrane transformation mechanics

### Fusion mechanics

Potential molecular mechanics underlying fusion pore opening have been reviewed repeatedly^1,8,16^. Here, we focus on fusion pore dynamics microscopically observed in real time.

#### Pore opening and expansion

Calcium binding with synaptotagmin induces SNARE proteins to mediate fusion in excitable cells^1,67^. Pore conductance and current measurements in secretory cells and SNARE-reconstituted nano-discs suggest that fusion machinery, including SNARE proteins, are pivotal in opening and expanding fusion pores at nanometer ranges^1,67–72^. STED imaging showed that cortical F-actin-supported membrane tension may expand fusion pores up to hundreds of nanometers (Fig. 2e)^40,73^.

#### Fusion pore constriction and closure

Early studies of vesicular spots implied dynamin in expanding^26,74,75^ or stabilizing fusion pores^75–77^. Recent studies showed that dynamin inhibition enhances quantal release, implying dynamin in controlling fusion pore^78^. STED imaging showed that dynamin inhibition blocks fusion pore constriction/closure, that dynamin is localized at fusion sites before fusion, and that dynamin scaffold surrounds and constricts Ω-profile’s pore in real time (Figs. 2e, 5cII)^40,79^.

How does dynamin know when to work? Calcium reduction or replacement with strontium blocked fusion pore constriction/closure, suggesting that calcium influx triggers fusion pore constriction/closure by activating dynamin (Fig. 2e)^39,40,54^. Since calcium-binding protein calmodulin, calcineurin, and protein kinase C may mediate calcium-triggered endocytosis^80–83^, they might activate dynamin by dephosphorylating and/or phosphorylating dynamin^80^, a proposal for future examination.

#### Fusion and fission machinery compete to decide pore dynamics

Since calcium influx may activate dynamin to mediate pore constriction and closure, a block of calcium influx or dynamin inhibits fusion pore constriction/closure and thus increases the initial pore size^39,40^. These effects can be antagonized by blocking F-actin- and tension-mediated fusion pore-expansion mechanisms, suggesting that dynamin-dependent pore constriction and SNARE/F-actin-dependent pore expansion compete to decide fusion pore and thus content release dynamics^39,40^. Unexpectedly, block of calcium influx or dynamin facilitates hemi-to-full fusion, suggesting that dynamin acts at the hemi-fusion stage before pore opening to compete with SNARE- and F-actin-dependent pore opening/expansion mechanisms^39,40^. In summary, dynamin-dependent pore constriction and SNARE/F-actin-dependent pore expansion compete to decide fusion dynamics, including hemi-to-full fusion, pore opening, expansion, constriction and closure (Fig. 2e). We suggest this competition scheme as a general principle governing fusion pore dynamics, where undiscussed (apology here) or future-learned mechanisms can be added to build a more complete model. Further supporting this principle, dynamic fusion pore expansion and constriction controlled by F-actin, myosin II and Bin-Amphiphysin-Rvs domain proteins were reported during micron-sized exocrine vesicle fusion^84^, and compared with smaller vesicles^85^.

#### Why shrink but not full-collapse fusion?

Experiments and realistic modeling suggest that a swelling osmotic pressure, the positive intra- to extracellular osmotic pressure difference, squeezes and thus abolishes membrane tension of the fusion-generated Ω-profile, producing a positive PM-to-Ω-profile tension gradient to reel the Ω-profile membrane into PM (Fig. 3dII)^41^. With squeezing and membrane-reeling-in force, Ω-profile shrinking is free energetically favored over full-collapse (Fig. 3d)^41^, explaining why full-collapse does not occur^2,7^. The PM-to-Ω-profile tension gradient depends on cortical F-actin, explaining why F-actin is crucial in mediating shrink fusion^41,73^. Since the swelling osmotic pressure and membrane-actin cortex adhesion required for shrink-fusion^41,73^ are general properties of the cell^73,86–89^, shrink-fusion may be of broad application.

The swelling osmotic pressure might contribute to shrinking micron-sized exocrine vesicles, which are compressed in minutes by a surrounding actomyosin network (Fig. 3aIII)^56,84^. This compression mechanism is too slow for rapid shrinking of much smaller endocrine vesicles^73^, but may work with the swelling osmotic pressure to underlie slower vesicle shrinking.

#### Rapid release site assembly at fused vesicles

Sequential compound fusion with a ~0.2-85 s interval suggests rapid assembly of release sites at the 1st fusing vesicle^42^. This finding suggests modifying the half-a-century concept—exocytosis occurs at preestablished release sites in excitable cells, such as active zones^1,2^—to include rapid release site assembly at fused vesicular Ω-profiles (Fig. 4c).

### Endocytosis mechanics

Tens of proteins and lipids have been implicated in mediating endocytosis using various curvature generation mechanisms, such as hydrophobic insertion, scaffolding, crowding, and liquid-liquid phase separation^3,90–94^. Here we focus on mechanisms underlying the flat→Λ→Ω→Ο transition observed in real-time.

#### Pulling and constriction underlie flat-to-round transition

During flat→Λ transition, Λ’s height increases with a spike-like membrane protrusion attached at Λ’s tip while Λ’s base remains constant and is surrounded by F-actin and dynamin scaffold (Fig. 5b, c; Supplementary Movies 8, 12, 13)^66,79^. Λ’s tip and its spike-like protrusion are associated with growing actin filaments and dynamin puncta (Fig. 5b, c; Supplementary Movies 8, 12, 13)^66,79^. Block of F-actin or dynamin inhibits flat→Λ transition^66,79^. These results suggest that F-actin and dynamin pull at the endocytic zone’s center and Λ’s tip while constraining the boundary from growing, leading to flat→Λ transition (Fig. 5d).

During Λ→Ω→Ο transition, the Λ-shape is converted into a Ω-shape as Λ’s base width decreases; dynamin puncta surround Λ’s base and Ω’s pore, and constrict in parallel with the constriction of Λ’s base and Ω’s pore (Fig. 5b–d; Supplementary Movie 13)^66,79^. Furthermore, block of dynamin inhibits Λ→Ω→Ο transition^66,79^. These results suggest that dynamin surrounds and constricts Λ-profile’s base as large as hundreds of nanometers, transforming Λ- to Ω-profile, and then constricts Ω-profile’s pore from up to hundreds of nanometers to zero, converting Ω-profiles to vesicles (Fig. 5d).

Mathematical modeling show that two forces, a pulling force at the center and a periphery-to-centre constriction force, are sufficient to mediate flat→Λ→Ω→Ο transition (Fig. 5d, Supplementary Movie 14)^79^. It is concluded that F-actin and dynamin mediate flat→Λ→Ω→Ο transition by pulling at the center and constriction at the base/pore region^79^.

#### F-actin pulls membrane inward

Imaging revealed that F-actin filament attached at the growing Λ-profile’s tip may pull Λ-profile inward (Fig. 5cI, Supplementary Movie 12)^79^. Modeling suggests that a ~3 pN point-pulling force is sufficient to pull membrane inward (Supplementary Movie 14)^79^. Such a small force might involve a bundle of contractile complexes of actin and myosin filaments with an anchored in the cytoplasmic actin network^95,96^. It differs from the proposal for yeast and mammalian clathrin-mediated endocytosis, where actin polymerization from PM to cytosol along the clathrin cage’s surface may generate pushing forces to elongate the bud^91,97–99^. Since STED imaging also visualized F-actin recruitment to growing Λ’s base and side (Fig. 5cI)^79^, both tip pulling and side pushing may contribute to Λ formation. These mechanisms may explain why F-actin inhibition by latrunculin A or actin β-isoform knockout reduces endocytosis and membrane pits at nerve terminals and inhibits clathrin-mediated endocytosis^79,86,100–102^.

#### Dynamin: master player of flat→Λ, Λ→Ω and Ω→Ο

While dynamin-mediated narrow (~5–20 nm) pore fission is well known^94,103^, imaging revealed surprisingly that dynamin is involved in pulling membrane inward with F-actin, and in mediating Λ→Ω→Ο by constricting Λ’s base and Ω’s pore as large as hundreds of nanometers (Fig. 5cII, d; Supplementary Movie 13)^79^. Supporting this finding, dynamin interacts with F-actin at actin comets, podosomes, filopodia, and F-actin bundles^103–106^. How dynamin interacts with F-actin to generate pulling forces remains to be studied.

Dynamin alone may surround and constrict large Λ’s base and Ω’s pore because dynamin forms helices surrounding and constricting liposomes from hundreds of nanometers down to the nanometer range^79^. Current models where dynamin helix conformational changes may constrict ~5-10 nm pore^94,103^ seem difficult to explain constriction of hundreds of nanometers. Other molecules might also be involved. Endophilin or synaptojanin 1 knockout increases membrane pits’ base at synapses, implying their involvement in neck formation^107^.

#### Endocytosis principle: two forces make one vesicle

Dynamin and actin are involved in most endocytic modes reported^2,100–102,108–111^. Accordingly, F-actin- and dynamin-dependent pulling and constriction that mediate flat→Λ→Ω→Ο transition may mediate many clathrin-independent but dynamin/actin-dependent modes of endocytosis, such as clathrin-dispensable but vesicle recycling-essential ultrafast, fast, slow, bulk, and overshoot endocytosis at synapses^2,9,100–102,108–114^, and clathrin-independent endocytosis of extracellular ligands, receptors, viruses, bacteria, prions, and bacterial toxins^90,115^. Even for clathrin-mediated endocytosis, dynamin and actin may exert their pulling and constriction forces to generate clathrin-coated pits. Supporting this possibility, initiation and maturation of fluorescent clathrin spots, which presumably involve pit formation, depend on dynamin and actin^91,92^; dynamin can be pre-recruited to endocytic zones via interaction with syndapin 1^116^; dynamin is located at the periphery of flat or shallow clathrin patches^117^, consistent with dynamin’s ability to constrict Λ-profile’s base^79^. We propose a general principle that F-actin- and dynamin-dependent pulling and constriction underlie flat-to-round transformation, regardless of the involvement of coat proteins like clathrin (Fig. 5d).

#### Calcium: unified trigger of flat→Λ, Λ→Ω and Ω→Ο

Calcium influx triggers diverse endocytic modes, including fast, slow, overshoot, and bulk endocytosis in secretory cells^2,81,83,118,119^ (but see Ref. ^120^). Calcium reduction or replacement with strontium blocks flat→Λ, Λ→Ω and Ω→Ο, suggesting calcium triggers each of these modular transitions^39,40,54,66^. By triggering flat→Λ, Λ→Ω and Ω→Ο, calcium influx initiates diverse endocytic modes, including slow, fast, ultrafast, overshoot, and bulk endocytosis^66^. Calmodulin, calcineurin and protein kinase C have been suggested as calcium sensors underlying calcium-triggered endocytosis by dephosphorylation and/or phosphorylation of endocytic proteins^80–83^. Synaptotagmin 1, an exocytosis calcium sensor, may also be involved in exo-endocytosis coupling^121–124^. These calcium sensors are candidates for underlying calcium-triggered flat→Λ, Λ→Ω and Ω→Ο transition.

#### Dynamin- and/or actin-independent endocytosis

Studies attempting to abolish dynamin or actin functions suggest the existence of dynamin- and/or actin-independent endocytosis. For example, applying the non-hydrolyzable GTP analog, GTP-γS, which abolishes the GTPase dynamin-mediated endocytosis, reveals dynamin-independent synaptic vesicle endocytosis^125^. Knockout of all three dynamin isoforms in fibroblasts uncovers a dynamin-independent fluid-phase endocytosis^126^. Results from the use of F-actin inhibitors (e.g., latrunculin A) are not all consistent^86,112,127–135^. A study suggests that actin is only needed at high-tension PM^86^, which may explain conflicting results from different but not the same preparations. Another study offers an alternative explanation that latrunculin A may not access certain actin pools^134^. Consistent with this explanation, synaptic vesicle endocytosis is inhibited by actin β or γ isoform knockout but not by latrunculin A^101,135^.

Actin’s role is minimized by the reduction of PM tension during clathrin-mediated endocytosis in yeast or mammalian cells, suggesting mechanisms other than F-actin in generating membrane invagination^86,136–138^. In brief, dynamin- or actin-independent mechanisms might contribute to underlying endocytic curvature transitions^3,93^.

#### Liquid-liquid phase separation

Liquid-liquid phase separation might contribute to endocytic curvature generation^93,138–143^. Eps15 and Fcho1/2 rely on weak, liquid-like interactions to promote the assembly of protein droplets in vitro, which may support clathrin-mediated endocytosis in vivo^139^. Similarly, Ede1, the yeast Eps15 homolog, may generate a separate liquid phase to nucleate endocytic patches^140^. Endophilin may undergo a phase transition into liquid-like condensates to facilitate endocytic protein assembly during fast endophilin-mediated endocytosis^141^. Dynamin 1 interacts with syndapin 1 to form molecular condensates for mediating ultrafast endocytosis at synapses^116^. Endocytic coat proteins with prion-like domains in yeasts may form hemispherical puncta with the hallmarks of biomolecular condensates^138^. Modeling suggests that cohesive interactions within condensates and interfacial tensions among condensates, membranes, and the cytosol might contribute to membrane invagination during actin-dependent and -independent endocytosis^138^. Testing these effects on real-time observed flat→Λ→Ω→Ο transition may pinpoint the exact curvature transition controlled by liquid-liquid phase separation.

## Functions

Exocytosis strength depends on many parameters, such as the vesicle release probability, release synchronization, single vesicle content release rate and amount, and availability of release sites^2,7,144^. Endocytosis efficiency depends on individual vesicle endocytosis’s speed, number and size. These key parameters are controlled by the exo-endocytosis membrane transformations. To give a bird’s view on this regulation, we synthesize a live-cell exo-endocytosis membrane transformation model (Fig. 6) based on recent real-time microscopic observations^39–42,54,66,73,79^. The transformation includes hemi-fusion, hemi-to-full fusion, fusion pore expansion, constriction, hemi-fission, hemi-to-full fission, fusing vesicular Ω-profile size enlargement or shrinking, sequential compound fusion and kiss-and-run, compound fusion (not being visualized), and endocytic flat→Λ→Ω→Ο transition (Fig. 6). These transformations offer many previously unrecognized or underappreciated mechanisms to control exo-endocytosis.

### Exocytotic functions

#### Hemi-fusion pathway defines vesicle release probability

Since a significant fraction of hemi-fusion structures fail to reach full fusion^39^, we suggest redefining release probability as hemi-fusion probability times hemi-to-full fusion probability (Fig. 6). Regulation of the hemi-fusion pathway may thus regulate release probability, underlying various forms of synaptic plasticity^145,146^.

#### A dynamic pore theory governing vesicular content release

Visualization of membrane dynamics established a dynamic pore theory: fusion pore may expand up to the vesicle width and then constrict and close while the fused vesicular Ω-profile size may remain unchanged, enlarge, shrink partially, or shrink completely (Fig. 6)^40,41,54^. This new model (Fig. 6) differs from the classical framework (Fig. 1d) in three aspects: (1) full-collapse fusion is replaced with shrink fusion or shrink-collapse fusion (Fig. 3c), (2) kiss-and-run is redefined to include any pore size that may limit or promote release and formation of different vesicle sizes due to enlarge-close or shrink-close fusion, and (3) shrink and enlarge fusion, rather than classical full-collapse and kiss-and-run, employ large and small pore to promote and limit content release, respectively^40,41,54^.

Decades of studies interpreted rapid/complete release, pore conductance increase beyond detection limit, or release of ~20 nm quantum dots as full-collapse fusion, and otherwise as kiss-and-run using the classical framework (Fig. 1d)^2,7,13,15–19,147^ may be subject to significant errors. We suggest replacing the classical framework with the new dynamic pore theory (Figs. 2e, 3c, 6) to account for content release. Shrink (or shrink-collapse) and enlarged fusion (Fig. 6) may mediate fast and slow content release previously attributed to full-collapse and kiss-and-run, respectively. Regulation of shrink and enlarge fusion might explain how modulators regulate content release^148–151^.

Redefined kiss-and-run may open a large or small pore to promote or limit release, respectively (Fig. 6). Partial content release has been implicated by measurements of catecholamine-mediated slow/small amperometric currents^13,152–154^, estimation of transmitter released fractions per vesicle^78,155^, and assessment of vesicular dopamine via correlative imaging with transmission EM and nanoscale secondary ion mass spectrometry^156^. These studies implied partial catecholamine release as the overwhelming majority^13,78,152–156^. In contrast, imaging neuropeptide Y release and fusion pore dynamics in chromaffin cells showed much infrequent partial release^40^. The reason for this apparent conflict is unclear.

Kiss-and-run is not the only mechanism underlying partial release because stay fusion may occasionally keep neuropeptide Y from release, likely via a pore narrower than neuropeptide Y’s molecular size^40^. Thus, partial release is due to pore constriction until it is narrower than the released content’s molecular size.

#### Compound fusion enhances quantal size and synaptic plasticity

Compound fusion releases vesicular contents equivalent to multiple regular vesicles, leading to an increase in the quantal size (Figs. 4c, 6). This increase contributes to underlying a common short-term synaptic plasticity induced by repetitive firings, the post-tetanic potentiation^23,157^.

#### Asynchronized release via hemi-fusion and sequential compound fusion

Vesicle release is mostly synchronized to relay fast presynaptic firings to postsynaptic neurons^158^. However, release can be asynchronized with various delays, which may enhance the dynamic range of neuronal impulse relay, synaptic plasticity, and neuromodulation^158^. Mechanisms underlying asynchronized release remain poorly understood^144^. Imaging revealed a delay of milliseconds to tens of seconds between hemi and full fusion and between 1st and 2nd fusion of a sequential compound fusion (Figs. 2b, 4b, 6)^39,42^. Slow hemi-to-full fusion or sequential compound fusion may thus generate asynchronized release. Their regulation may modulate synchronized versus asynchronized release.

#### Sequential compound fusion enhances exocytosis capacity

Repetitive stimulation induces many fused vesicular Ω-profiles that may occupy and thus block release sites. Likely to overcome this problem, sequential compound fusion takes place at these Ω-profiles to enhance exocytosis capacity, and to save vesicles from traveling one-vesicle-length distance for docking at original release sites (Fig. 6)^42^.

### Endocytic functions

#### Preformed Ω-profile closure and kiss-&-run underlie diverse endocytic modes

In excitable cells of the nervous or endocrine system, where exocytosis may deplete vesicles during repetitive firing, endocytosis must recycle fused vesicles rapidly to sustain exocytosis^2,9^. To meet this demand, cells employ various endocytic modes, including speed-specific slow (>~6 s), fast (<~6 s) or ultrafast (<~0.6 s) endocytosis, amount-specific compensatory (endocytosis = exocytosis) or overshoot (endocytosis > exocytosis) endocytosis, and size-specific bulk endocytosis (forming large endosome-like structures) or clathrin-mediated endocytosis (forming small vesicles)^2,4,9,22,81,100,159–165^. Many of these modes are also observed in non-excitable cells^91,115,166,167^.

These endocytic modes are thought to undergo flat-to-round transformation using different yet largely unclear molecular mechanisms to achieve their specificity^2,24,166,168^. Historically, fast endocytosis was considered too fast to be mediated by slow clathrin-mediated endocytosis^2^. Kiss-and-run was thus hypothesized, although not at consensus, to underlie fast endocytosis^2,24^. Ultrafast endocytosis is thought to undergo a flat-to-round transformation using endocytic machinery containing dynamin, actin, endophilin, and syndapin, but not clathrin^100,107,112^, and with dynamin primed at the endocytic site^116^. Pre-enrichment of endophilin might also contribute to generating fast endophilin-mediated endocytosis^166,169^.

The known differences in the molecular machinery seem difficult to explain different endocytic modes satisfactorily. Studies of real-time-observed membrane transformations offer a sound explanation for generating endocytic modes differing in speeds, amounts, and vesicle sizes with the same molecular machinery. In chromaffin cells, brief depolarization may induce ultrafast, fast, slow, compensatory, overshoot, and/or bulk endocytosis, as recorded with whole-cell capacitance measurements (Figs. 2a, 5e)^54,66^. The depolarization also induces exo-endocytic membrane transformation detected with imaging, including fusion pore opening and closure, flat→Λ→Ω→Ο, preformed-Λ→Ω→Ο and preformed-Ω→Ο transition^54,66^. Summing these membrane transitions yielded a reconstructed exo-endocytosis trace matching the capacitance-recorded exo-endocytic mode from the same cell^54,66^. Quantification revealed surprisingly that preformed-Ω→Ο and fusion pore closure (kiss-and-run), but not flat→Λ→Ω→Ο, are the primary mechanism underlying each endocytic mode, including ultrafast, fast, slow, compensatory, overshoot, and bulk endocytosis (Figs. 5e, 6)^66^. Preformed-Ω→Ο and kiss-and-run each contributes about half to each endocytic mode, except overshoot caused mainly by preformed-Ω→Ο^66^.

The only difference among these modes is the calcium current^66^, the trigger of each endocytic membrane transformation^39,40,54,66^, and each endocytic mode^2,81,118,170^. The calcium current increases in order as the capacitance-recorded endocytic mode changes from no-endocytosis to slow, fast, ultrafast, and overshoot endocytosis^54,66^. By controlling preformed-Ω and fusion pore closure’s speed, number, and vesicle size, low to high calcium influxes generate in order (1) slow, fast, and ultrafast endocytosis; (2) no-endocytosis, compensatory, and overshoot endocytosis; and (3) regular- and large-sized (bulk) vesicle endocytosis (Fig. 5e)^54,66^. Thus, with the same endocytic machinery, different amounts of the trigger signal, calcium influx, may generate endocytic modes differing in speeds, quantities, and vesicle sizes^66^.

These findings may explain the long-held mystery of how a ‘slow’ flat-to-round machinery produces fast/ultrafast endocytosis—by closing preformed or fusion-generated Ω-profiles that are fission-ready^66^. It explains the longstanding conundrum that secretory vesicle endocytosis measured after depolarization is much faster than receptor-mediated endocytosis measured from the entire flat-to-round transition. Preformed Ω-profile closure explains long-held mysteries at synapses that ‘readily retrievable’ vesicular proteins, but not newly exocytosed ones, are retrieved first^171–173^, and that a readily retrievable membrane pool is retrieved upon depolarization^174^. Preformed Ω-profile may constitute the readily retrievable membrane pool. Given that kiss-and-run^2,13,15,17,18^, preformed Ω-profiles, and endocytosis overshoot that reflects preformed Ω-profile closure are widely observed in endocrine cells, neurons, and beyond^4,73,81,100,101,160,164,175,176^, the new concept that preformed Ω-profile closure and kiss-and-run underlie diverse endocytic modes may have broad application. While emphasizing this new concept, the contribution of flat-to-round transition can be more significant if the low probability flat→Λ→Ω→O transition is enhanced in specific conditions or cell types yet to be visualized.

#### Controlling vesicle sizes

Vesicle size and transmitter concentration determine the quantal size in secretory cells^23,177–180^. Vesicular contents may regulate vesicle size via unknown mechanisms^178,179^. Imaging revealed five manners membrane transformations control vesicle size: (1) shrink-close fusion generates smaller vesicles, (2) enlarge-close fusion forms larger vesicles^41,54^, (3) sequential compound fusion followed by 1st fusing vesicle pore closure produces large vesicles^42^, (4) compound fusion generates large vesicles^23,63,64,181^, and (5) Λ- and Ω-profile may grow to different extents during flat→Λ→Ω→Ο transition, resulting in different vesicle sizes^66^ (Fig. 6). Shrink-close^41,54^ and sequential compound fusion^42^ may create elongated vesicles due to compression by the swelling osmotic pressure^41^, explaining elongated vesicles observed with EM^2,4,165,182^. These mechanisms may explain vesicle size changes caused by protein manipulations. For example, dynamin’s role in flat→Λ→Ω→Ο^79^ may explain why dynamin 1 controls synaptic vesicle size^183^. Regulating these mechanisms might underlie synaptic plasticity, as compound fusion contributes to mediating post-tetanic potentiation^23^.

### Exo-endocytosis coupling

Exocytosis is followed by endocytosis often with a similar amount^2^. Two mechanisms may mediate such an exo-endocytosis coupling: (1) calcium influx that triggers both exo- and endocytosis^2,81,83,118^ (but see ref. ^120^), and (2) SNARE proteins that mediate exocytosis are also required for mediating endocytosis^184–186^. Membrane tension, which regulates endocytosis^187^, could be another potential coupling factor if exocytosis can change membrane tension^188^.

Imaging revealed that calcium influx triggers each membrane transformation, including fusion, fusion pore closure, flat→Λ, Λ→Ω and Ω→O transition^40,54,66^. Varying calcium influxes from low to high levels increase (1) the speed of preformed Ω-profile closure and fusion pore closure, generating speed-specific slow, fast and ultrafast endocytosis; (2) the probability of preformed Ω-profile closure and fusion pore closure, generating amount-specific no-endocytosis, compensatory and overshoot endocytosis, and (3) vesicle size, generating size-specific endocytic modes like bulk endocytosis^66^. Thus, while triggering more intense exocytosis, larger calcium influx triggers faster and more endocytosis by inducing preformed Ω-profile closure and fusion pore closure at higher speeds and quantities, explaining how calcium influx couples exo- to endocytosis with a matched intensity (Fig. 5e).

## Concluding remarks, future challenges and opportunities

Based on real-time observed membrane transformations, we synthesize a new model (Fig. 6) replacing the classical framework of fusion and budding (Fig. 1d). In this new model, fusion involves hemi-fusion, hemi-to-full fusion, fusion pore expansion, constriction, hemi-fission, and hemi-to-full fission, while the fusing vesicular Ω-profiles may maintain its size, enlarge, shrink partially or completely, or become a new release site for sequential compound fusion; endocytosis involves modular Flat→Λ, Λ→Ω and Ω→O transition, each with a low transition probability (Fig. 6). Compound fusion may also occur. Kiss-and-run is redefined as rapid or slow closure of any size of fusion pores, which may generate different sizes of vesicles; full-collapse fusion is replaced with shrink fusion; a shrink-collapse fusion is proposed to reconcile the debate between shrink and full-collapse fusion.

Many mechanistic principles underlying fusion and budding have been suggested. First, competition between fusion and fission machinery determines fusion membrane dynamics, vesicle release probability, synchronized versus asynchronized release, vesicular content release rates/amounts, and quantal size. Furthermore, compound fusion also regulates quantal size, underlying synaptic post-tetanic potentiation. Second, cells’ swelling osmotic pressure and cytoskeleton-dependent membrane tension underlie fusing vesicle size changes, particularly shrink fusion. Third, rapid-release site assembly at fused Ω-profiles enables sequential compound fusion to enhance exocytosis capacity. Fourth, F-actin/dynamin-dependent pulling at the endocytic zone center and a dynamin-mediated constriction from the periphery are sufficient to mediate Flat→Λ→Ω→O transition. Fifth, preformed and fusion-generated Ω-profile pore closure, but not flat-to-round transition, primarily underlie all kinetically distinguishable endocytic modes, including ultrafast, fast, slow, bulk, compensatory, and overshoot endocytosis. Sixth, with the same endocytic machinery, low to high levels of the endocytosis trigger signal, the calcium influx, generates in order (1) speed-specific slow, fast, and ultrafast endocytosis, (2) amount-specific no-endocytosis, compensatory, and overshoot endocytosis, and (3) vesicle size-specific regular- and large-sized (bulk) vesicle endocytosis by controlling performed and fusion-generated Ω-profile pore closure’s speed, number, and vesicle size. Seventh, calcium influx couples exo- to endocytosis by triggering each exo-endocytosis membrane transition—larger calcium influx induces more intense exocytosis and a matched intensity of endocytosis. These principles may be conserved for fusion and budding in various cells and organelles.

The techniques developed for real-time visualization of membrane transformations^40,66^ open the door to examine fusion, budding, and, more generally, the curvature generation in live cells. However, time-lapse STED imaging was from single XZ-planes (Box 1)^40,66^, making data collection especially time-consuming^189,190^. A STED volume scan could help solve this problem, but it is practically too slow to catch fast events and may strongly bleach fluorophores^189,190^. Techniques with faster scanning, less bleaching, brighter labeling, and higher detection sensitivity are needed to make super-resolution volume scanning practical^191–194^. To resolve membrane transformation of small vesicles like synaptic vesicles and clathrin-coated vesicles, it requires a spatial-temporal resolution of ~10 nm or smaller at sub-second or even millisecond resolution. Developing such a microscopic technique seems extremely challenging, but is ultimately necessary for real-time visualization of small vesicles.

The model in Fig. 6 is in its early stage with many open questions, as dynamic protein structural changes at a nanometer-millisecond scale that underlie each membrane transformation are largely unclear. For example, it remains unclear how dynamin competes with SNARE proteins for the limited nanodomain to antagonize hemi-to-full fusion and pore expansion, how release sites are rapidly assembled at fused Ω-profiles to support sequential compound fusion, how F-actin works together with dynamin to pull membrane inward, how dynamin constricts large Λ-profiles’ base and Ω-profiles’ pore, how SNARE proteins open the fusion pore, how dynamin mediates hemi- and then hemi-to-full fission, how clathrin and other membrane coat proteins contribute to Ω-profile formation, how calcium influx triggers each exo-endocytic membrane transformation, how tens of exo-endocytosis proteins and lipids not discussed here contribute to each exo-endocytic membrane transformation, and how the principles summarized here apply to different cell types or organelles. Addressing these questions in the future will provide a molecular understanding of membrane fusion and budding. With the synthesized model as the backbone (Fig. 6), we suggest adding future-learned knowledge to this backbone to understand fusion and budding comprehensively.

### Supplementary information

Description of Additional Supplementary Files

Supplementary Movie 1

Supplementary Movie 2

Supplementary Movie 3

Supplementary Movie 4

Supplementary Movie 5

Supplementary Movie 6

Supplementary Movie 7

Supplementary Movie 8

Supplementary Movie 9

Supplementary Movie 10

Supplementary Movie 11

Supplementary Movie 12

Supplementary Movie 13

Supplementary Movie 14
