# Supplementary material for: Membrane transformations of fusion and budding
Source: Nat Commun. 2024 Jan 2;15:21. doi: 10.1038/s41467-023-44539-7 (PMC10761761; doi:10.1038/s41467-023-44539-7)
Supplement: Supplementary file 1 — Description of Additional Supplementary Files [file 41467_2023_44539_MOESM1_ESM.pdf]

Description of Additional Supplementary Files

File name: Supplementary Movie 1

Description: Hemi-fusion. Hemi-fusion: PH_G-labelled Ω-shape profile (green) is not accompanied with uptake of Atto 532 (A532, red) from the bath solution to the Ω-profile. Green: PH_G-labelled plasma membrane; red: A532-labelled extracellular solution. The chromaffin cell was stimulated with a 1-s depolarization and images were acquired with the STED microscopy at the XZ plane on the cell-bottom plasma membrane (green), underneath which is a thin layer of bath solution and a coverslip (not visible) below the extracellular solution (applies to Movies 1-13). Horizontal length of the image frame is 1.0 μm. The Movie (from Ref. [39]) is played at real-time.

File name: Supplementary Movie 2

Description: Hemi-fusion and then hemi-to-full fusion. Hemi-to-full fusion: PH_G-labelled Ω-profile (green, labelling cytosol-facing leaflet of the Ω-profile) appeared first, followed by filling of bath A532 (red) into the Ω-profile. PH_G: PH-EGFP; horizontal length of the image frame is 1.3 μm; the Movie (from Ref. [39]) is played at real-time.

File name: Supplementary Movie 3

Description: Close-fusion (kiss-and-run) with a large pore. Direct visualization of fusion pore opening and closure. Green: PH_G-labelled plasma membrane; red: A532-labelled extracellular solution; green and red channels are superimposed at the bottom. PH_G-labelled Ω-profile pore closure was accompanied by A532 spot dimming, which verified the pore closure that prevented bleached A532 (strongly excited) inside the closed vesicle from exchanging with bath fluorescent A532. Horizontal length of the image frame is 1.3 μm; the Movie (from Ref. [40]) is played at real-time.

File name: Supplementary Movie 4

Description: Shrink-fusion. Fusion-generated PH_G-labelled Ω-profile (green) and A532 spot (red) shrink until not visible (green and red channels are superimposed at the bottom). Horizontal length of the image frame is 1 μm; the Movie (from Ref. [41]) is played at real-time.

File name: Supplementary Movie 5

Description: Enlarge-fusion. Fusion-generated PH_G-labelled Ω-profile (green) and A532 spot (red) enlarge after fusion (green and red channels are superimposed at the bottom). Horizontal length of the image frame is 1.6 μm; the Movie (from Ref. [41]) is played at real-time.

File name: Supplementary Movie 6

Description: Sequential compound fusion. Green: PH_G-labelled plasma membrane; red: A532-labelled extracellular solution; green and red channels are superimposed at the bottom. Horizontal length of the image frame is 1.4 μm; the movie (from Ref. [42]) is played 4 times as fast as the real-time.

File name: Supplementary Movie 7
Description: Sequential compound release. Sequential compound fusion causes sequential compound release of fluorescent false neurotransmitter FFN 511 preloaded in vesicles (sequential release from two vesicles). Green: PH_G-labelled plasma membrane; Magenta: fluorescent false neurotransmitter FFN 511; both channels are merged at the bottom. Horizontal length of the image frame is 1.2 μm; the movie (from Ref. [42]) is played 2 times as fast as the real-time.

File name: Supplementary Movie 8
Description: Flat→Λ transition. Green: PH_G-labelled plasma membrane; red: A532-labelled extracellular solution; green and red channels are superimposed in the lower panel. Horizontal length of the image frame is 1.8 μm; the Movie (from Ref. [66]) is played at real-time. Data taken from Ref. [66].

File name: Supplementary Movie 9
Description: Λ→Ω transition. Green: PH_G-labelled plasma membrane. Horizontal length of the image frame is 1.7 μm; the Movie (from Ref. [66]) is played at real-time.

File name: Supplementary Movie 10
Description: Ω→O transition. Green: PH_G-labelled plasma membrane; red: A532-labelled extracellular solution; green and red channels are superimposed in the lower panel. Horizontal length of the image frame is 1.6 μm; the Movie (from Ref. [66]) is played at real-time.

File name: Supplementary Movie 11
Description: Flat→Λ→Ω→O transition. Green: PH_G-labelled plasma membrane; red: A532-labelled extracellular solution; green and red channels are superimposed in the lower panel. Horizontal length of the image frame is 1.3 μm; the Movie (from Ref. [66]) is played at 3 times as fast as the real-time.

File name: Supplementary Movie 12
Description: Actin filament in the act of pulling membrane inward to form Λ-shape profile. Green: PH_G-labelled plasma membrane; red: Lifeact-labelled F-actin; green and red channels are superimposed in the lower panel. Horizontal length of the image frame is 1.7 mm; the Movie (from Ref. [79]) is played at real-time.

File name: Supplementary Movie 13
Description: Dynamin in the act of constricting Λ-profile's base and Ω-profile's pore. Green: PH_G-labelled plasma membrane; red: dynamin 1-mTFP1 puncta. green and red channels are superimposed in the lower panel. Horizontal length of the image frame is 1.7 μm; the Movie (from Ref. [79]) is played 2 times as fast as the real-time.

File name: Supplementary Movie 14
Description: Simulated shape evolution for the Flat→Λ→Ω transition. Evolution of simulated 3-D shapes for Flat→Λ and Λ→Ω transitions combined together. The values of the pulling force, f_{pull}, and the base radius, r_b, are shown for each frame. All lengths are scaled by the intrinsic length, r_i. Increasing the pulling force, f_{pull}, while constraining the base to a constant value, $r_b =$

$2.5 \cdot r_i$, results in Flat to Λ-shape transition. The shape transformation continues smoothly from Λ-shape to Ω-shape by the base constriction upon conservation of the pulling force ($f_{pull} \cong 7.2 - 7.3\ f_i$) and membrane area. Movie is from Ref. [79].
